# Free Final Time Input Design Problem for Robust Entropy-Like System Parameter Estimation

**DOI:** 10.3390/e20070528

**Published:** 2018-07-14

**Authors:** Wiktor Jakowluk

**Affiliations:** Faculty of Computer Science, Bialystok University of Technology, Wiejska 45A, 15-351 Bialystok, Poland; w.jakowluk@pb.edu.pl; Tel.: +48-85-746-91-12; Fax: +48-85-746-97-22

**Keywords:** free final time, input signal design, model fitting, optimal control, parameter estimation

## Abstract

In this paper, a novel method is proposed to design a free final time input signal, which is then used in the robust system identification process. The solution of the constrained optimal input design problem is based on the minimization of an extra state variable representing the free final time scaling factor, formulated in the Bolza functional form, subject to the D-efficiency constraint as well as the input energy constraint. The objective function used for the model of the system identification provides robustness regarding the outlying data and was constructed using the so-called Entropy-like estimator. The perturbation time interval has a significant impact on the cost of the real-life system identification experiment. The contribution of this work is to examine the economic aspects between the imposed constraints on the input signal design, and the experiment duration while undertaking an identification experiment in the real operating conditions. The methodology is applicable to the general class of systems and was supported by numerical examples. Illustrative examples of the Least Squares, and the Entropy-Like estimators for the system parameter data validation where measurements include additive white noise are compared using ellipsoidal confidence regions.

## 1. Introduction

System identification is typically carried out by perturbing processes or plants under operation and use experimental data to construct the model of the dynamic system. The main objective is to find a set of dynamic models that describe important properties of the true system [1]. The fundamental task in system identification is to excite the system of interest using an informative input and build the model of the system with maximum pertinence [2,3]. The problem of the optimal input signal design is typically solved by minimizing an a priori selected norm of the Fisher information matrix with respect to an appropriate experimental setup [4]. The prediction error methods (PEM) are a wide collection of the parameter estimation methods that minimize a weighted norm of the prediction error [2]. The identification experiment can be executed in both closed and open loop conditions and could be utilized for arbitrary model parameterizations. Improper experiment conditions can cause performance degradation of the control loop. It has been reported that about 80% of the designed control loops do not guarantee the acceptable performance assessment [5].

That is why some authors introduced the idea of the performance degradation minimization instead of the variance minimization of the estimated parameters. The robust control identification considers the uncertainty of the estimated model on the designed closed-loop system performance [6]. System identification for robust control allows comparing the performance of the unknown real system loop with a controller tuned using an identified plant model. The least-costly identification experiment for control, where the main goal is to design an experiment that ensures a small enough uncertainty region but still provides an acceptable performance of control, was proposed in [7,8]. It was found that, during the advanced control loop designs, model construction absorbs approximately 75% of the costs [9].

The plant-friendly input design is classified as the application-oriented methodology. The aim of such an identification experiment is to find a trade-off between the minimal disruption to the normal operation of the system, and the most precise identification experiment [10,11]. There have been some reports that plant friendliness constraints often disturb a precise model parameters estimation while a set of harmonically related sinusoids with high peak-to-peak values can destroy an identified model [3,12]. For this reason, safer excitation signals which ensure more precise model identification are presented in [13,14]. The issue of optimal input design in the economic framework, where the cost based on plant friendly constraint is minimized, was developed in [15]. One of the current trends in accordance with application-oriented input design is the use of the model predictive control (MPC) technique [16,17]. This formulation is based on the input design procedure to obtain an acceptable control performance that still provides revealing data for system identification [18]. The main idea is to choose the spectral density of an input signal that ensures that estimated parameters are acceptable while the experimental cost is minimized. For an overview of application-oriented input design in application to system identification, see the survey [19]. In the exact model identification for control purposes, the optimal experiment design methods should be considered. For this reason, Entropy-based optimal experiment design method for discrimination between competing models was presented in [20]. The approach of model discrimination was based on the expected Shannon entropy reduction of the Bayesian model weights uncertainty.

Considering the automatic control aims and objectives for model identification, the outlying data have a critical impact on model parameters to be estimated. The most utilized prediction error estimator methods are Least Squares (LS) and Weighted Least Squares (WLS) where the sum of the squared residuals is minimized [21]. Different robust formulations [22] where the penalty function is minimized with respect to the overall distribution of residuals are Least Median of Squares (LMS), Least Trimmed Squares (LTS), and Reweighted Least Squares (RLS). The Maximum Likelihood (ML), Minimum Entropy (ME), and Generalized Maximum Entropy (GME) approaches for robust parameter identification, which guarantee robustness subject to regression models, were presented in [23]. The novel prediction error parameter estimation method named Least Entropy-Like (LEL) estimator was developed in [24]. This algorithm is based on correctly established penalty function and was built according to the Gibbs entropy definition. In a previous paper [25], the spectrum approximation issue based on the idea of optimal prediction was presented. The THREE-like approach minimizes a divergence factor subject to a given spectral density. The contribution of this work was to define a new divergence family which compares two spectral densities with respect to an optimal prediction task. In this approach, the output covariance of a bank of filters was utilized to obtain information on the input spectrum power. The interpretation of the dual problem of the THREE-like approach is shown as a new parametric spectral approximation problem wherein the optimal result of the spectral density is closest to the correlogram. It has been shown that two particular THREE-like solutions are equivalent to the prediction error identification method [26]. In the above paper, an important connection between time and spectral domain entropy rates was presented in details.

After a brief review of current literature, there is still an interest in parameter identification from real-life data [27]. The methodology presented in this paper can be successfully used in continuous and discrete-time state-space model identification including linear and nonlinear models with higher orders. In a previous paper [28], a first-order linear time-invariant system case study of plant friendly input signal design with respect to the D-efficiency was presented. The economical and plant friendly input design for linear discrete-time system identification, where the goal was to minimize the cost incurred during the experiment with respect to plant friendly constraints, can be found in [15]. Numerical results for second-order linear torsional spring system identification were introduced in [29]. The optimal input design problem for parameter estimation in a nonlinear water tank system through a model output sensitivity minimization subject to its parameters was solved in [30]. However, all the existing works in the literature consider the optimization methods of designing the excitation signals with fixed final time conditions only. In contrast, the present article discusses the formulation and the solution scheme of a free final time optimal input design problem. For this purpose, the state-space equation is augmented by an extra state variable representing free terminal time scaling factor (i.e., according to [31]). The contribution of this study is to use the free terminal time inputs to examine the economic aspects between the imposed constraints on the input signal shape, and the parameter estimates while undertaking a robust identification experiment utilizing an implementation of the LEL algorithm. The constraints imposed on input signal solution should allow one to attain a slight information loss from the plant whose operating point is disturbed in the safest way.

This paper is structured as follows. In Section 2, the problem statement of the constraint optimal input design for parameter estimation of a system is described. The Least Entropy-Like estimator for the robust system identification is presented in Section 3. In Section 4, D-optimal input signal design and the transcription for free final time problem is derived. The problem reformulation for free final time constraint input design is shown in Section 5. The results of simulation experiments for a linear time-invariant model case study are presented in Section 6. Finally, concluding comments are made in Section 7.

## 2. Problem Statement

Consider below system described as:

(1) y(t)=f(u,t,θ,v), 

where *y(t)*, *u(t)*, *v(t)* and *θ* are, respectively: output, input, noise, and parameter of the system. System identification is the process of building an accurate dynamic mathematical model of a system from experimental data and a priori plant knowledge. The precision of the model parameter estimates depends on the selection of an optimal perturbation signal. In the design of optimal input signals for a plant model parameters calculation, an appropriate scalar norm of the Fisher information matrix (FIM) must be chosen as the objective criterion. The FIM is defined as follows:

(2) $$M=E\left[\frac{\partial }{\partial \theta }\ln p\left(y|\theta \right){\left(\frac{\partial }{\partial \theta }\ln p\left(y|\theta \right)\right)}^{T}\right].\;$$

The D-optimality norm for which determinant of the Fisher information matrix *detM* is maximized or *det*(*M*^−1^) is minimized is often used. Chosen measures of optimal design performance could be found in [32]:

- A-optimality (*tr*(*M*^−1^)) minimizes the total variance of the parameters estimates.
- E-optimality (*λ*_max_(*M*^−1^)) minimizes the variance of the maximum eigenvalue of *M*^−1^.
- D-optimality minimizes the generalized variance of the parameters and minimizes the volume of the ellipsoidal confidence region of parameter estimates with respect to the input.

To obtain optimal input signal, an unbiased estimator of *θ* should be considered. In this case, the covariance of the parameter estimates is given by the Cramer–Rao inequality, viz., the inverse of the FIM. Then, the covariance of the estimate $\hat{\theta }$ can be defined as:

(3) $$\mathrm{cov}\left(\hat{\theta }\right)=E\left[\left(\hat{\theta }-\theta \right){\left(\hat{\theta }-\theta \right)}^{T}\right]\geq M{}^{-1}\;$$

The single weighted cost function method for time domain input signal design was presented in a previous paper by the author [28]. While the choice of the experiment norm is essential, the inputs designed based on some performance criteria may not be suitable for the plant excitation [2]. The input signal used for a plant model excitation should, at the same time, fulfill two requirements: the acceptable precision of the model parameter estimates and the system should be perturbed in the safest way. These conditions can be met using an approach, which is based on the notion of the D-efficiency [28]. The D-efficiency is often expressed as a percentage scale and may be considered as a measure of the sub-optimality of any synthesized input signal:

(4) ED(e)=100%{det(M(e))det(M(e*))}1k, 

where *k* denotes the number of parameters to be identified, and e* indicates the D-optimal design. Regarding the analysis presented in *t* [28], we impose the inequality scalar constraint on the D-efficiency formulation. In general, the optimal input design task is formulated through maximization of the FIM determinant and should take into account the equality and inequality constraints imposed on the conditions. The experiment performed in such a way should yield safe behavior of the perturbation signal. Some of these constraints could be defined as:

- D-efficiency inequality constraint can be expressed according to (4).
- Amplitude constraints on inputs, outputs or state variables in a form:
  (5) $$u_{\min}\leq u(t)\leq u_{\max}.\;$$
- Energy constraints imposed on inputs:
  (6) $$\int_{0}^{T}u^{T}\left(t\right)u\left(t\right)dt\leq E.\;$$

The goal of this study was to design the constraint input signal with free final time conditions, which is then used to the robust identification experiment. In this case, the performance index is formulated through the minimization of the free terminal time scaling factor, subject to the D-efficiency constraints as well as input energy constraints. In that way (i.e., by taking into account such a set of constraints to optimal input design), we can obtain the suboptimal input signal, which is safer for system identification purposes.

## 3. System Identification Method

In system identification for control purposes, outlying data have a critical impact on plant model development including precise parameter estimation. In this study, the least squares and the novel Entropy-Like estimators were compared for plant model parameters identification purposes. For these reasons, the basic concept of the prediction error method for system identification is presented in this section. The algorithm is based on the specific objective function minimization with respect to prediction error residuals. The concept of the cost function formulation was motivated by the Gibbs entropy definition. A more detailed overview of the Entropy-Like method (LEL) was presented in [24].

To solve the optimization task presented above, the following system was considered:

(7) $$\begin{array}{l} y_{i}=f\left(x_{1},x_{2},\ldots ,x_{l},\theta_{r}\right)+\varepsilon_{i},\\ i=1,2,\ldots ,l, \end{array}\;$$

where *θ_r_* is a parameter vector to be estimated, *y_i_* is the output samples sequence, and *ε_i_* is the Gaussian white noise with restricted variance. The model prediction error estimators are obtained utilizing the regression residuals:

(8) $$r_{i}=y_{i}-{\hat{y}}_{i},\;$$

where ${\hat{y}}_{i}$ are the approximated outputs, i.e., ${\hat{y}}_{i}=f\left(x_{1},x_{2},\ldots ,x_{l},\hat{\theta }\right).$ The Least Squares method (LS) for regression analysis is standard and very popular approach. The performance index minimizes the sum of squared residuals of the fit (i.e., prediction error estimator) is:

(9) $${\hat{\theta }}_{LS}=\mathrm{Arg}\underset{\theta }{\min}\sum_{i=1}^{N}r_{i}^{2}=\mathrm{Arg}\underset{\theta }{\min}\left(r^{T}r\right),\;$$

where *r* is the residual vector of the fit $r=f\left(r_{1},r_{2},\ldots ,r_{N}\right).$ A different algorithm, which is robust for model parameter identification, is based on penalty function minimization.

The Least Entropy-Like estimator (LEL) was developed utilizing the concept of Gibbs entropy. The main objective of this method is to analyze the global dispersion measure of the residuals fit. The prediction error estimator built according to Equation (8) is as follows:

(10) $$S=\sum_{j=1}^{N}r_{j}^{2},\;$$

the comparative squared residuals can be presented as:

(11) $$\mathrm{if}\;S\neq 0\text{​​​ }\mathrm{then}\;s_{i}=\frac{r_{i}^{2}}{\sum_{j=1}^{N}r_{j}^{2}}\;\mathrm{where}\;s_{i}\in \left[0,1\right],\sum_{i=1}^{N}s_{i}=1,\;$$

according to the reasoning presented in [24], the cost function *Φ* based on normalized entropy was chosen as:

(12) $$\Phi =\left\{ \begin{array}{l} 0\;\mathrm{for}\;S=0\\ -\frac{1}{\log N}\sum_{i=1}^{N}s_{i}\log s_{i}\;\mathrm{for}\;S\neq 0 \end{array}\right..\;$$

Then, the Least Entropy-Like (LEL) estimator defined as a measure of dispersion of the relative squared rest values is given by:

(13) $${\hat{\theta }}_{LEL}=\mathrm{Arg}\underset{\theta }{\min}\Phi ,\;$$

Entropy-like formulation (12) affects the values of the unknown parameters *θ* through the predictive error residuals. The LEL estimator (13) is robust with respect to outliers because the objective function is minimized subject to relative squared errors variability. It could be noticed that the cost function *Φ* is nonlinear and may not provide the unique minimum subject to the unknown parameters *θ*. Considering the basic algorithm properties, one should initially examine the Least Squares quality fit. As presented in [24], the LEL estimator can be numerically computed from an initial condition value close to the real parameter value. In the experimental part of this paper, the LS and the novel LEL estimators would be compared for plant model parameters identification purposes.

## 4. Optimal Input Design Problem

In the paper, the problem of synthesizing the constraint optimal input with free terminal conditions for system identification is considered. The general idea is to define a nominal period [0, *T_f_*] and to replace free final time with fixed final time problem utilizing a scaling factor as an augmented state variable, which scales the duration of the time interval. We solve this problem using the transcription of the below optimal control formulation into a similar optimal control task represented in the Lagrange form with the set of constraints. To verify the suitability of this technique to the model parameter identification, a first-order time-invariant inertial system is considered:

(14) $$\begin{array}{l} \dot{x}\left(t\right)=ax\left(t\right)+bu\left(t\right),\;x\left(0\right)=x_{0},\\ y\left(t\right)=x\left(t\right)+v\left(t\right), \end{array}\;$$

where *x*(*t*) is a state variable, *u*(*t*) is a control function, *y*(*t*) is a measurement, *a* and *b* are constant model parameters and *v*(*t*) is a zero mean Gaussian white noise process as follows:

(15) $$\begin{array}{l} E\left[v\left(t\right)\right]=0,\\ E\left[v\left(t\right)v^{T}\left(\tau \right)\right]=R\delta \left(t-\tau \right)=\sigma_{n}^{2}\delta \left(t-\tau \right). \end{array}\;$$

The fundamental principle of system parameter estimation is to maximize the sensitivity of the state variable to the unidentified parameters [1]. The motivation for such an approach is the Cramer–Rao definition, which gives a lower bound for the variance of an unbiased parameter to be estimated. Applying the above definition to input design purposes, we calculate the parameter estimate which has a tendency of getting lower for optimal input:

(16) $$\mathrm{cov}\left(\left[a,b\right]\right)\geq M^{-1}.\;$$

The FIM for the inertial state-space model (14) can be formulated as follows:

(17) $$M\left(T\right)=\int_{0}^{T}X_{\theta }^{T}R^{-1}X_{\theta }dt=\frac{1}{\sigma_{n}^{2}}\int_{0}^{T}\left[\begin{array}{c} x_{a}\\ x_{b} \end{array}\right]\left[\begin{array}{cc} x_{a} & x_{b} \end{array}\right]dt=\frac{1}{\sigma_{n}^{2}}\int_{0}^{T}\left[\begin{array}{cc} x_{a}^{2} & x_{a}x_{b}\\ x_{a}x_{b} & x_{b}^{2} \end{array}\right]dt,\;$$

where *x_a_ =* ∂*x/*∂*a*, *x_b_ =* ∂*x/*∂*b*, and *R* is 2 × 2 matrix given by:

(18) $$R^{-1}=\left[\begin{array}{cc} R^{-1} & 0\\ 0 & R^{-1} \end{array}\right]=\frac{1}{\sigma_{n}^{2}}\left[\begin{array}{cc} 1 & 0\\ 0 & 1 \end{array}\right].\;$$

Substituting Equation (17) into Equation (16) yields:

(19) $$\mathrm{cov}\left(\left[a,b\right]\right)\geq \frac{\sigma_{n}^{2}}{M\left(T\right)}.\;$$

In the considerations which follow, it was assumed that *σ_n_* = 1 to obtain an optimal input signal for system parameters identification where measurements do not include additive white noise. To maximize the FIM determinant, let us define the augmented state vector given by [1]:

(20) $${\dot{x}}_{a}=x+ax_{a},\;x_{a}\left(0\right)=0,\;$$

(21) $${\dot{x}}_{b}=ax_{b}+u,\;x_{b}\left(0\right)=0.$$

The Fisher information matrix to the inertial model (14) has the following form:

(22) $$M\left(t\right)=\left[\begin{array}{cc} m_{11}\left(t\right) & m_{12}\left(t\right)\\ m_{21}\left(t\right) & m_{22}\left(t\right) \end{array}\right].\;$$

To design an optimal input signal with free terminal time conditions, the optimal control problem solver RIOTS_95 was utilized [33]. The Matlab toolbox Riots allows solving a very large class of finite-time optimal control problems that includes: trajectory and end-point constraints, variable initial conditions, free final time tasks and problems with cost functions endpoint. The objective function to be minimized can be formulated in Bolza form as:

(23) $$J=g\left(x\left(t_{0}\right),x\left(t_{f}\right)\right)+\int_{t_{0}}^{t_{f}}l\left(x,u,t\right)dt,\;$$

in respect to the plant dynamics, and with the initial condition:

(24) $$\dot{x}(t)=h\left(x,u,t\right),\text{​​ }x\left(t_{0}\right)=x_{t_{0}},t\in \left[t_{0},t_{f}\right],\;$$

subject to the constraints:

(25) $$u\left(t\right)\in \langle u_{\min}\left(t\right),u_{\max}\left(t\right)\rangle ,t\in \left[t_{0},t_{f}\right],\;$$

(26) $$x\left(t_{0}\right)\in \langle x_{\min}\left(t_{0}\right),x_{\max}\left(t_{0}\right)\rangle ,\;$$

(27) $$l_{tc}^{\varsigma }\left(t,x\left(t\right),u\left(t\right)\right)\leq 0,\varsigma \in q_{\mathrm{tc}},t\in \left[t_{0},t_{f}\right],\;$$

(28) $$g_{eic}^{\varsigma }\left(x\left(t_{0}\right),x\left(t_{f}\right)\right)\leq 0,\varsigma \in q_{\mathrm{eic}},\;$$

(29) $$g_{eec}^{\varsigma }\left(x\left(t_{0}\right),x\left(t_{f}\right)\right)=0,\varsigma \in q_{\mathrm{eec}}.\;$$

where *x* is the state-space vector, *t* ∈ [*t*_0_, *t_f_*] denotes time duration, q = {1, …, *q*} and *l*, *g*, and *h* are *a priori* linear or nonlinear functions. The functions *g*(·,·) and *l*(·,·,·) with indexes *tc*, *eec*, and *eic* are trajectory constraint, endpoint equality constraint and endpoint inequality constraint, respectively.

The free terminal time problem can be included in the form of an optimal control problem by augmenting the state equations by further state variables, i.e., one extra state for each independent problem. According to the reasoning presented in Section 2 of the user manual [33], the general idea is to define a nominal time interval [0, *T_f_*] and to replace the free final time problem with the fixed final time case utilizing the free final time scaling factor as an augmented state variable, which scales the duration of the time interval. For this reason, the scale factor and the scaled time are expressed by extra states which enables minimization over initial value of the further states to fit the scaling.

Assuming that the state space differential equation is described by:

(30) $$\dot{x}=h\left(t,x,u\right),\;$$

the cost function has the following form:

(31) $$J=g\left(x\left(T\right)\right)+\int_{0}^{T}l\left(t,x,u\right)dt,\;$$

where x(*t*) is the state space vector, u(*t*) is the state input vector, and *T* denotes the free terminal time of the experiment.

Including two extra state variables *x_n+_*_1_ and *x_n+_*_2_ to (30), the free terminal time problem can be modified into the similar fixed final time optimal control problem with an augmented state vector:

(32) $$\left(\begin{array}{c} \dot{x}(t)\\ {\dot{x}}_{n+1}\\ {\dot{x}}_{n+2} \end{array}\right)=\left(\begin{array}{c} x_{n+2}h\left(x_{n+1,}x,u\right)\\ x_{n+2}\\ 0 \end{array}\right),\;$$

where *t* ∈ [0, *T_f_*] and the objective function can be written as:

(33) $$J=g\left(x\left(x_{n+2}T_{f}\right)\right)+\int_{0}^{T_{f}}l\left(x_{n+1},x,u\right)dt,\;$$

where *t* ∈ [0, *T_f_*], *x_n+_*_2_ is the duration scale coefficient to be minimized, *x_n+_*_1_ = *tx_n+_*_2_ denotes free termination time, and *T_f_* is the fixed termination time chosen arbitrarily. When considering the autonomous dynamic systems, the extra state variable *x_n+_*_1_ is not obligatory. Therefore, the autonomous free final time problem can be solved by augmenting state equations considering only one state variable representing the free final time scaling factor.

## 5. Problem Reformulation

As described in [29] with reference to the single weighted cost function method, the derivations for inertial system dynamics are given below. The augmented state Equations (20) and (21), and the FIM matrix elements containing an extra state variable representing free final time scaling factor *ζ* are as follows:

(34) $$\begin{array}{lll} x_{1}=x; & {\dot{x}}_{1}=x_{7}\left(ax_{1}+bu\right); & x_{1}\left(0\right)=x_{10};\\ x_{2}=x_{a}; & {\dot{x}}_{2}=x_{7}\left(x_{1}+ax_{2}\right); & x_{2}\left(0\right)=0;\\ x_{3}=x_{b}; & {\dot{x}}_{3}=x_{7}\left(ax_{3}+u\right); & x_{3}\left(0\right)=0;\\ x_{4}=m_{11}; & {\dot{x}}_{4}=x_{7}x_{2}^{2}; & x_{4}\left(0\right)=0;\\ x_{5}=m_{12}=m_{21}; & {\dot{x}}_{5}=x_{7}x_{2}x_{3}; & x_{5}\left(0\right)=0;\\ x_{6}=m_{22}; & {\dot{x}}_{6}=x_{7}x_{3}^{2}; & x_{6}\left(0\right)=0;\\ x_{7}=\zeta ; & {\dot{x}}_{7}=0; & x_{7}\left(0\right)=\zeta_{70}. \end{array}$$

The proposed method can be applied to a general class of systems given by the state-space representation of any order. Finally, the optimal control problem based on Bolza canonical formulation, which minimizes the objective function (23) with respect to D-efficiency equality constraint (4) as well as input (25) and trajectory (27) constraints, is:

(35) $$J\left(u\right)=\zeta +q\int_{0}^{T}u{\left(t\right)}^{T}u\left(t\right)dt,\;$$

with respect to:

(36) $$\begin{array}{c} -1\leq u(t)\leq 1,\;t\in \left[0,T\right],\;\\ 0.1\leq \zeta \leq 10,\\ x_{1}\left(t\right)\leq 1,\;t\in \left[0,T\right],\\ \left(-x_{4}\left(T\right)x_{6}\left(T\right)+x_{5}^{2}\left(T\right)\right)=D, \end{array}\;$$

where *D* is the desired D-efficiency constant, *q* is an input energy factor and *ζ*_70_ is an initial state condition to be optimized. It should be noted that an additional constraint was imposed on the state variable *x*_1_(*t*) to enable unexpected changes of the control signal *u*(*t*), which is restricted to the interval [−1, +1]. Using the proposed methodology defined by Equations (34)–(36), the optimal solution for free final time is *t_f_* = *Tζ*.

## 6. Numerical Results

To solve the issue presented above, the RIOTS_95 toolbox [33] for solving optimal control problems can be adapted. This software is implemented in Matlab as a separate module and has tools for solving constrained optimal control problems with fixed or free final time conditions.

Constrained optimal inputs for the first-order, linear time-invariant (LTI) model of the system were then computed for the assumed initial values of parameters: *a* = −1, *b* = 1, and nominal time duration *t* = [0, 10] s, using sequential quadratic programming (SQP) algorithm. The initial state conditions of the inertial model were selected to be *x*_1_(0) = 5, *x*_7_(0) = 1, and the initial value of the input signal was set as *u*(0) = 1. The free final time scaling factor *ζ* which scales the duration of the time is optimized from the interval 0.1 ≤ *ζ* ≤ 10, so the time duration could be varied from 1 to 100 s. The numerical results were computed using the fixed step-size fourth-order Runge–Kutta integration method with grid period of 0.2 s. The expression for the cost function, given by Equation (35), can be presented as:

(37) $$J\left(u\right)=J_{1}+qJ_{2},\;$$

where *J*_1_ denotes the free final time scaling factor *ζ,* and *J*_2_ is the integral of the squared input signal.

### 6.1. Free-Final Time Constraint Input Design

**Figure 1:**
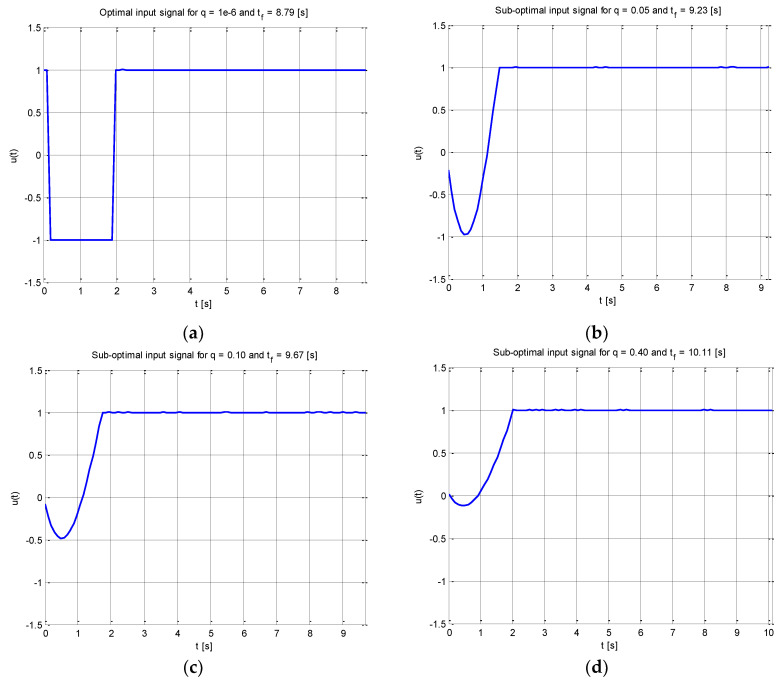
Free final time inputs to inertial model perturbation: (**a**) optimal input signal for *q* ≈ 0 and *D*_eff_ = 90%; (**b**) suboptimal input signal for *q* = 0.05 and *D*_eff_ = 90%; (**c**) suboptimal input signal for *q* = 0.10 and *D*_eff_ = 90%; and (**d**) suboptimal input signal for *q* = 0.40 and *D*_eff_ = 90%.

The D-optimal input signal received when there was no constraint on the input energy value (i.e., the coefficient *q ≈* 0 in the equality (35) is displayed in Figure 1a. It corresponds to the D-optimal experiment *e*, where the desired value of the FIM determinant is obtained (according to (4)) in such a manner that *D*_eff_ = 90% of its optimal value. The suboptimal input signals obtained for different desired values of the input energy factor *q* and D-efficiency constant value *D*_eff_ = 90% are displayed in Figure 1c,d.

The D-optimal perturbation signal received when there was no constraint on the input energy component (i.e., for *J*_1_ = 0.88, *qJ*_2_ = 1.0 × 10^−4^ and *t*_f_ = 8.79 [s]) is shown in Figure 1a. The input energy factor was increased (Figure 1b) to obtain the suboptimal input signal, which corresponds to performance index components values: *J*_1_ = 0.92, *qJ*_2_ = 5.00 and *t*_f_ = 9.23 [s]. For comparison, Figure 1c shows the suboptimal input signal, which correlates with the objective function values: *J*_1_ = 0.97, *qJ*_2_ = 8.70 at the final time level of *t*_f_ = 9.67 [s]. Figure 1d contains the graphical display of the suboptimal excitation received for the cost function integrants values: *J*_1_ = 1.01, *qJ*_2_ = 33.44, where time duration was *t*_f_ = 10.11 [s].

As we can see, when the desired value of input energy factor increases, the shape of the optimal excitation considerably changes. While for the optimal experiment (in the sense of Equation (4)) there are the rapid changes of the input, the control signals obtained for *D*_eff_ < 100% are safer for system identification purposes until the FIM determinant is not dominated by the input energy component of the minimized performance index. The comparison of the performance index components obtained for increasing values of the input energy factor and for decreasing values of D-efficiency from the interval [100%, 80%] of its maximum value are presented in Table 1, Table 2 and Table 3.

As could be noticed based on the presented method (see Table 1, Table 2 and Table 3), when the desired value of the input energy factor increases, the D-optimal signal duration also increases. When the required value of the D-efficiency from the interval [100%, 80%] decreases, considered signals duration also decreases. As we can see (Table 1), the optimal input signal duration for inertial system identification (i.e., *J*_1_ = 1, *q* = 1 ×10^−6^ and *D*_eff_ = 100%) is equal to 10 s.

### 6.2. LS and LEL Estimators for LTI Model Identification

The D-optimal input signals *u*(*t_f_*), computed as solutions of the free final time optimization problems (35) and (36), were then utilized as excitations in the plant model parameter estimation procedure. The physical system (14), used in system identification procedure, can be described by the following single input–single output state space model:

(38) $$\begin{array}{l} {\dot{x}}_{1}\left(t_{f}\right)=ax_{1}\left(t_{f}\right)+bu\left(t_{f}\right)+v\left(t_{f}\right),\;x_{1}\left(0\right)=x_{10},\\ y\left(t_{f}\right)=x_{1}\left(t_{f}\right), \end{array}\;$$

**Figure 2:**
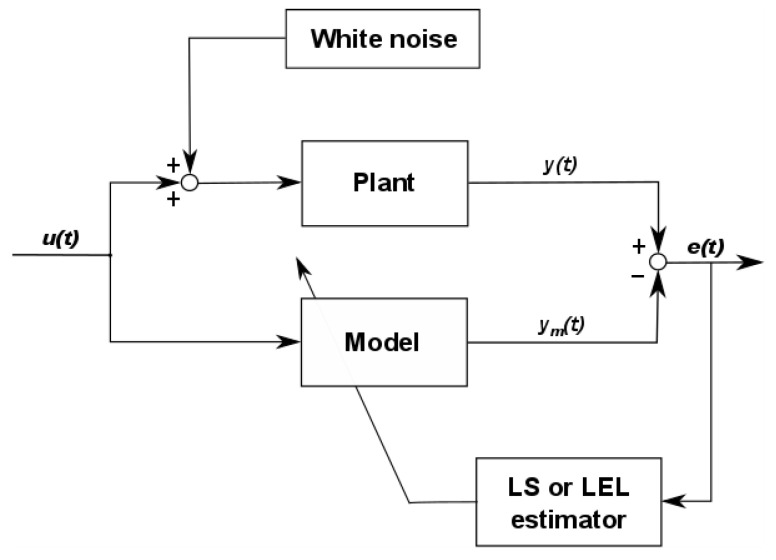
The system parameter identification block diagram.

The scheme shown in Figure 2 presents the process of the plant model parameter estimation: we excite the system input using *u*(*t_f_*), and we collect data on its output *y*(*t_f_*).

A disturbance signal with different variance from the interval 0.0 ≤ *σ*^2^ ≤ 0.7 is added to the control input to the system. The model of the plant (38) depends on a vector of unknown parameters *θ* = [*a*, *b*]^T^ and the aim of such an experiment is to estimate unknown model parameters values which should be the most similar to the true values of the plant parameters. The difference between the output of the plant *y*(*t_f_*) and the output of the model *y_m_*(*t_f_*) was minimized. The initial state condition of the inertial model was selected from the interval −5 ≤ *x*_1_(0) ≤ 5 and the experiment duration depends on chosen D-optimal signal according to Table 2. Numerical results were obtained utilizing the Nelder–Mead simplex method.

**Figure 3:**
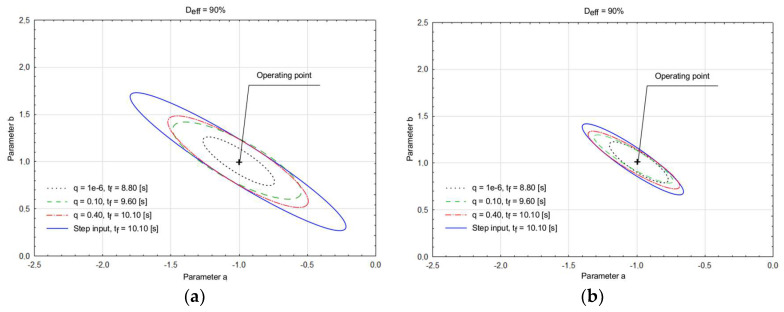
Ellipsoidal confidence regions of LS parameter estimates (**a**); and LEL parameter estimates for different inputs: D-optimal input signal (black dotted, *D*_eff_ = 90%, *q* = 1 × 10^−6^), Suboptimal input signal (green dashed, *D*_eff_ = 90%, *q* = 0.10), Suboptimal input signal (red dash-dotted, *D*_eff_ = 90%, *q* = 0.40), and Step input signal (blue solid line) (**b**).

The distribution of the model parameters *a* and *b* obtained as the results of the optimization tasks using conventional LS and robust LEL estimators with different control inputs are shown in Figure 3.

Eighty-eight computations were done when the plant model starts from various initial state condition, and the additional noise disturbing the system input has a different variance. Figure 3a contains the ellipsoidal confidence region (black dotted line) with the input signal obtained for the minimal value of input energy factor (i.e., *q* ≈ 0 and *D*_eff_ = 90%), while Figure 3b shows the results (for the same values of initial states and noise variance) with excitation signals that were computed when an input energy coefficient increases its value and was selected as *q* = 0.10 (green dashed line) and 0.40 (red dash-dot line). To compare results, Figure 3 contains the graphical display of the ellipsoidal confidence region of parameter estimates, where the system (Figure 2) was perturbed utilizing a step input signal (blue solid line). The comparison of the ellipsoidal confidence regions of the plant model parameter estimates indicates some similarities. The D-optimal input signal, calculated for *q* ≈ 0, causes the minimal time duration of the parameter identification experiment and a minimal volume of the ellipsoidal confidence region of parameter estimates. When the value of the input energy factor increases, the area under the curve increases its size for the same initial conditions and noise variance values. Increasing the desired ratio of input energy constraint yields the increase in the input signal duration, but the excitation is safer for the plant. In such a manner, we avoid rapid switching of the excitation signal in the real identification experiments. The prediction error LS, and the relative squared error LEL estimators were compared based on maximum, average, and minimum residuals of the parameter values (Table 4 and Table 5).

**Figure 4:**
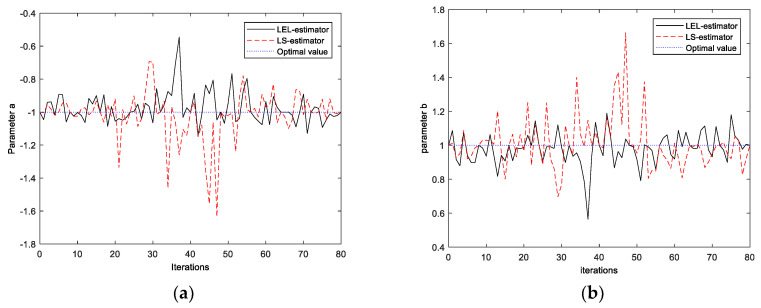
The comparison of the estimates values obtained using LEL (black solid line), and LS (red dashed line) estimators for various initial conditions, different noise variance, and D-efficiency value 90%: (**a**) confrontation between rival estimators for parameter estimate *a* based on the 80 runs; and (**b**) confrontation between rival estimators for parameter estimate *b* based on the 80 runs.

As could be noticed based on Table 4 and Table 5, a properly implemented penalty function of the LEL algorithm yields, in most cases, more precise estimates for different values of the energy constraint, D-efficiency constraint, and the experiment duration. The comparison of the estimates for parameters *a* and *b* is shown in Figure 4.

The function *Φ* defined by Equation (12) depends on parameters *a* and *b* by means of relative squared residuals. The main idea of the LEL estimator (13) is to make most of the residuals striving for zero value or to make the relative squared residuals equally distributed according to minimization criteria. Data points related to large residuals are called the outlying data points. The proposed method is robust with respect to outliers because the cost function to be minimized is a measure of the relative squared errors variability. In the above simulation experiment, the outlier data occurred for inertial system initial condition *x*_1_(0) = 1. The reason for this obstruction is the excitation signal (i.e., unit step-like input) which is not able to unbalance the inertial system with respect to the initial condition equal to 1. Therefore, data outlier points were removed from the set of the parameter estimates. Finally, it should be noticed that there is no guarantee for relative squared residuals formulation to have a unique solution with respect to the parameters. Thus, the minimization should be performed very carefully with special attention given to the initialization of the parameters.

## 7. Discussion

The novelty of this work was to design the free final time input signals, with constraints on input move size and D-efficiency value which are then used in the system identification experiments. The objective of such an experiment was to minimize the extra state variable representing the free final time scaling factor, subject to a carefully selected set of constraints. Some of these signals obtained as the solution of the free final time optimization task were then used as inputs in the plant model parameter estimation procedure. The parameter identification experiments were performed in the presence of a white noise affecting the system input. The imposed constraints, which quantify the plant trajectories, allow one to obtain safer excitation signals while still providing acceptable confidence regions of the plant model parameter estimates.

Another objective of this work was to find the relationships between the imposed constraints on the input signal design and the experiment duration in the real-time parameter identification experiments. The results of the above experiments confirm our assumption that the input signal obtained for input energy factor value q ≈ 0, yields the minimal duration of the identification experiment and a minimal volume of the ellipsoidal confidence region of parameter estimates. When the D-efficiency constraint percentage ratio decreases, then the experiment duration is reduced as well as the economic cost of identification under normal operation. However, safer input signals obtained for different required values of the input energy factor yield the relative accuracy degradation of the plant model parameter estimates (observed as an increased volume of the confidence ellipsoid regions).

An identification cost for industrial purposes is difficult to obtain because it must contain actual costs during the regular production process. The signals used to system perturbation or resulting outputs of the identified system can affect other production processes. In the presented method, the cost is quantified in terms of departure from the nominal operational policy. During the closed loop identification experiment, the framework discussed in this paper is utilized to design a reference signal instead of the input signal to the open-loop model. The solution of the identification task appears to be robust with respect to the initial conditions variation and was expressed as a non-convex problem.

It was shown that applying the proposed method to system identification purposes yields an interesting empirical solution. The results obtained in the identification experiments prove that there is a trade-off between the experiment duration (i.e., closely related to input signal duration) and the accuracy of parameter estimates which depends on the friendliness of the input signal to be used. Thus, the cost of the identification experiment should be considered either as the experiment duration or as a measure of the performance deterioration.

## Figures and Tables

**Table 1:** Comparison of the performance index components for the *D*_eff_ = 100%.

*D*_eff_/*D*_opt_	FIM	*J*_1_	*q*	*qJ*_2_	*t_f_* [s]
100%	−43.87	1.00	1 × 10^−6^	1 × 10^−4^	10.00
		1.06	0.05	4.79	10.57
		1.10	0.10	8.99	11.00
		1.13	0.20	17.42	11.36
		1.15	0.30	25.91	11.53
		1.16	0.40	34.44	11.62
		1.17	0.50	42.97	11.70

**Table 2:** Comparison of the performance index components for the *D*_eff_ = 90%.

*D*_eff_/*D*_opt_	FIM	*J*_1_	*q*	*qJ*_2_	*t_f_* [s]
90%	−35.60	0.880	1 × 10^−6^	1 × 10^−4^	8.79
		0.923	0.05	5.00	9.23
		0.967	0.10	8.70	9.67
		0.991	0.20	16.86	9.91
		1.041	0.30	25.14	10.04
		1.011	0.40	33.44	10.11

**Table 3:** Comparison of the performance index components for the *D*_eff_ = 80%.

*D*_eff_/*D*_opt_	FIM	*J*_1_	*q*	*qJ*_2_	*t_f_* [s]
80%	−28.10	0.763	1 × 10^−6^	1 × 10^−4^	7.63
		0.807	0.05	4.35	8.07
		0.837	0.10	8.27	8.37
		0.858	0.20	16.25	8.58
		0.866	0.30	24.22	8.66

**Table 4:** The percentage accuracy rates for different values of *q*, and *D*_eff_ = 90%.

Index	*q* = 1 × 10^−6^				*q* = 0.10			
Estimator	LS		LEL		LS		LEL	
Parameters	*a*	*b*	*a*	*b*	*a*	*b*	*a*	*b*
average value [%]	9.08	8.99	6.01	6.57	10.97	11.75	8.05	8.68
maximum value [%]	61.27	63.54	47.54	40.44	55.69	57.82	40.74	42.18
minimum value [%]	2.0 × 10^−2^	1.6 × 10^−1^	2.1 × 10^−4^	3.3 × 10^−4^	1.9 × 10^−1^	7.0 × 10^−2^	1.5 × 10^−4^	1.2 × 10^−4^

**Table 5:** The percentage accuracy rates for different values of *q*, and *D*_eff_ = 90%.

Index	*q* = 0.40				Step Input Signal			
Estimator	LS		LEL		LS		LEL	
Parameters	*a*	*b*	*a*	*b*	*a*	*b*	*a*	*b*
average value [%]	12.29	12.45	9.02	9.49	14.35	14.23	9.83	10.56
maximum value [%]	63.27	47.43	49.98	43.15	83.19	86.95	54.49	59.76
minimum value [%]	4.6 × 10^−2^	9.6 × 10^−1^	1.3 × 10^−6^	9.5 × 10^−5^	1.9 × 10^−1^	2.3 × 10^−2^	4.0 × 10^−4^	1.5 × 10^−4^
